# Identification of technology frontiers of artificial intelligence-assisted pathology based on patent citation network

**DOI:** 10.1371/journal.pone.0273355

**Published:** 2022-08-22

**Authors:** Ting Zhang, Juan Chen, Yan Lu, Xiaoyi Yang, Zhaolian Ouyang

**Affiliations:** Institute of Medical Information & Library, Chinese Academy of Medical Sciences and Peking Union Medical College, Beijing, People’s Republic of China; Hanyang University, REPUBLIC OF KOREA

## Abstract

**Objectives:**

This paper aimed to identify the technology frontiers of artificial intelligence-assisted pathology based on patent citation network.

**Methods:**

Patents related to artificial intelligence-assisted pathology were searched and collected from the Derwent Innovation Index (DII), which were imported into Derwent Data Analyzer (DDA, Clarivate Derwent, New York, NY, USA) for authority control, and imported into the freely available computer program Ucinet 6 for drawing the patent citation network. The patent citation network according to the citation relationship could describe the technology development context in the field of artificial intelligence-assisted pathology. The patent citations were extracted from the collected patent data, selected highly cited patents to form a co-occurrence matrix, and built a patent citation network based on the co-occurrence matrix in each period. Text clustering is an unsupervised learning method, an important method in text mining, where similar documents are grouped into clusters. The similarity between documents are determined by calculating the distance between them, and the two documents with the closest distance are combined. The method of text clustering was used to identify the technology frontiers based on the patent citation network, which was according to co-word analysis of the title and abstract of the patents in this field.

**Results:**

1704 patents were obtained in the field of artificial intelligence-assisted pathology, which had been currently undergoing three stages, namely the budding period (1992–2000), the development period (2001–2015), and the rapid growth period (2016–2021). There were two technology frontiers in the budding period (1992–2000), namely systems and methods for image data processing in computerized tomography (CT), and immunohistochemistry (IHC), five technology frontiers in the development period (2001–2015), namely spectral analysis methods of biomacromolecules, pathological information system, diagnostic biomarkers, molecular pathology diagnosis, and pathological diagnosis antibody, and six technology frontiers in the rapid growth period (2016–2021), namely digital pathology (DP), deep learning (DL) algorithms—convolutional neural networks (CNN), disease prediction models, computational pathology, pathological image analysis method, and intelligent pathological system.

**Conclusions:**

Artificial intelligence-assisted pathology was currently in a rapid development period, and computational pathology, DL and other technologies in this period all involved the study of algorithms. Future research hotspots in this field would focus on algorithm improvement and intelligent diagnosis in order to realize the precise diagnosis. The results of this study presented an overview of the characteristics of research status and development trends in the field of artificial intelligence-assisted pathology, which could help readers broaden innovative ideas and discover new technological opportunities, and also served as important indicators for government policymaking.

## 1. Introduction

Artificial intelligence (AI) is considered to be one of the core driving technologies of the fourth industrial revolution. Since the 21st century, AI has made a series of breakthroughs. With the improvement of algorithm, computing power, computer hardware and the advent of the era of big data, AI has flourished and gradually penetrated into the medical industry, revolutionizing the traditional medical model. AI plays an important role in disease assessment and diagnosis, bringing great convenience to clinical work. AI could use sophisticated algorithms to “learn” features from massive amounts of medical data, and then use the insights gained to aid clinical practice. It can also be equipped with learning and self-correction capabilities to improve its accuracy based on feedback [1]. The subfields of AI include machine learning (ML), natural language processing (NLP), computer vision, etc. ML is a branch of AI, which mainly studies how computers simulate or realize human learning behavior, acquire new knowledge, improve existing knowledge framework and its own performance. The traditional ML algorithms include logistic regression, linear regression, decision tree (DT), naive bayes (NB), random forest (RF), support vector machine (SVM), multi-layer perceptron (MLP) and so on. In the medical field, ML could be applied to cluster patient characteristics, infer the probability of disease outcomes, etc [2]. ML could perform tasks without explicit programming instructions, discover hidden relationships between data, and perform data analysis. However, traditional ML needs to extract features from raw data and process them into structured data sets, which could not directly process unstructured data. Deep learning (DL) could directly process unstructured data including images, sounds, and languages, which has advantages in clinical image classification, medical record text analysis, and tumor diagnosis. DL was proposed by Hinton in 2006, which is a method to build multi-layer neural networks on unsupervised data features, a branch of ML. It is capable of automatically learn various features in the data, avoiding manual feature selection. DL mainly includes generative adversarial network (GAN) for data enhancement, recurrent neural networks (RNN) and recursive neural network (R-NN) for text analysis and NLP, and convolutional neural network (CNN) for image processing, etc. CNN is a representative algorithm of DL, which has unique advantages in image processing and has been used for feature extraction and analysis of clinical image data. CNN has two major characteristics, it could effectively reduce the dimensionality of pictures with large amounts of data into small amounts of data, and also retain image features and conform to the principles of image processing. The CNN algorithm is able to effectively process the image data of tumor for segmentation, internal feature extraction, and classification. The image processing includes image acquisition, image preprocessing, image segmentation, feature extraction, classification and recognition, etc., and ML algorithm is involved in every link. The image segmentation process uses clustering, CNN and other algorithms. Algorithms involved in feature extraction compromise of CNN, genetic algorithm, SVM, clustering, etc. Classification and recognition use CNN, MLP, SVM, DT, NB, logistic regression, etc. ML algorithms learn from many diagnosed samples collected from medical test reports in company with the experts’ diagnoses to support medical experts in predicting and diagnosing diseases in the future. The use of ML can assist in enhancing the reliability, performance, and accuracy of diagnosing systems for specific diseases.

Pathology plays an important role in disease diagnosis and treatment. 70% of medical diagnoses depend on the pathology, which is considered to be the “gold standard” for disease diagnosis. There are many objective problems in the pathology industry. It takes more than ten years to train a mature pathologist. The workload of pathologists is very large, and the regional differences in pathological medical resources cannot meet the needs of a large number of pathological diagnosis in primary hospitals. And their workloads have also increased due to the growing cases and the need to provide optimal treatment options from a wider range of diagnoses. There is a growing shortage of pathologists. The breakthroughs in AI are already having a major impact around the world. There has been an exponential growth in the application of AI in pathology. The integration of AI will be a milestone for health care in the next decade, and pathology is right at the focus of this revolution. The innovation of AI technologies could transform diagnostic pathology [3], which makes it possible of earlier disease detection, more precise and quantitative diagnosis, discovery of new contexts in human biology, and progress on personalized diagnostics and patient care [4]. Pathology is now recognized as a strong candidate for AI development, principally in the field of cancer diagnosis and tissue biomarker analytics. This has been driven primarily by the development of whole slide imaging (WSI) platforms and digital pathology (DP). Here, the generation high resolution digital images, each of which carries high volumes of data capturing the complex patterns, are critical to disease diagnosis, providing a fertile opportunity to apply AI for improved detection of disease [5, 6]. An attractive application area of AI is the analysis of histopathology, which currently requires careful evaluation of gigapixel-sized images by dedicated physicians and pathologists. Using AI technology, through training and learning a large number of confirmed pathological pictures, it can quickly realize intelligent recognition of pathological images, thereby greatly improving the efficiency and accuracy of pathological diagnosis [7, 8].

Patents cover more than 90% of the latest technological information in the world, with the detailed and accurate content. Patent analysis is one the theories and methods of obtaining intelligence from patent information, and it is one main method for analyzing the key technologies in information science [9, 10]. Through the patent analysis in a certain field, it can objectively reflect the overall situation and development trend of technology, which could effectively and accurately give the information about the relevant technological innovation and development [11, 12]. Patent citation analysis is one of the important means of patent analysis. The citation relationship between patents could highlight the scientific basis of the technology, and point out the pre- and post-inheritance and accumulation relationship between technologies. Therefore, based on patent citation network, it could describe the technology development path of a specific field, and provide important reference for the evaluation, selection and estimation of technology. There have been many related studies on patent citation analysis. The method of patent citation network has been successively applied to identify the technology development track in different fields, such as medical knowledge [13], fuel cells [14], and 3D printing technologies [15]. Peter et al. used the patent citation network to discover the process of technology innovation, and identify and predict technology convergence and clustering [16]. Kim et al. identified key technologies in the process of technology fusion through patent citation network [17]. Manajit et al. provided a perspective of citation network analysis of patents from a statistical viewpoint [18]. Zhong et al. explored technology evolution paths and identified key technology frontiers based on a patent co-citation analysis [19]. A citation network is an information network that represents the topical relevance. Through the patent citation network, it could map the correlation between technologies, and measure the characteristics and influence of technologies. The patent citation network established based on the citation path could show the evolution direction of related technologies more intuitively.

The importance of pathology to disease diagnosis cannot be underestimated, and precise pathological diagnosis has become one of the main bottlenecks affecting the development of precision medicine. In the context of big data, AI based on DL is increasingly influencing the diagnostic modalities of pathology. The application of AI in pathology provides an opportunity for further realization of precision medicine. A clear diagnosis is the core part of tumor diagnosis and treatment, and it is not only a hot spot but also a difficulty that needs to solved in AI pathology at present. And pathological features extracted based on AI could help guide the selection of treatment options. AI-assisted pathology are developing rapidly and are the focus of international competition. Through preliminary research, it was found that more than 25,000 scientific papers had been published in the field of AI-assisted pathology in Web of Science (WOS), and there were many researches in the fields of oncology and neuroscience. In the field of neuroscience, it is primarily concerned with Alzheimer’s disease (AD). The current hot papers focus on COVID-19 and breast cancer, all of which are related to DL. The main affiliations include Harvard University, University of California, University of London, University of Texas, National Institute of Health and Medical Research (INSERM), National Center for Scientific Research (CNRS), etc., each with more than 400 publications, mainly focusing on DL, hidden markov models (HMM), neural networks, etc. Judging from the published papers, there were very few papers about patent analysis of AI pathology, with only one systematic review so far. The review was published on Cancers on 13 May 2022, which is a systematic evaluation of the patent landscape on DP, a branch of pathology. This review is a patent analysis of AI in DP, which assesses the application and publication trends, major assignees, and leaders in the field, and expects that the patent application will focus on the digitization of pathological images and AI technologies that support the critical role of pathologists [20]. At present, no articles on patent analysis of the entire field of AI-assisted pathology has been retrieved. It is very meaningful to carry out a comprehensive patent analysis in this field, which could enrich the knowledge composition of this field. Patents are very important research objects and the most effective carriers of technological information. Moreover, 70% ~ 80% of technological inventions are only disclosed through patent documents, so that patents are the most suitable objects for technology analysis research, which could display the technological innovation in a field comprehensively. Therefore, our study selects patents of AI-assisted pathology as the research object, and identifies the technology frontier through patent citation analysis, which could help us grasp the trend and direction of technology innovation. This study could provide information support for the research of pathology, and also provide a new research perspective for technology development.

## 2. Materials and methods

### 2.1 Ethics statement

This study is about the patent analysis of AI-assisted pathology, and the subject investigated are the patents. It does not involve human subject research and animal research, and no patient records/information and clinical records are included. Therefore, no ethics issue is involved in this research.

### 2.2 Materials

Patents of AI-assisted pathology were collected from the Derwent Innovation Index (DII, 1981-present) of Clarivate Analytics (formerly Thomson Reuters). DII is one of the world’s largest patent document database developed by Clarivate Analytics, covering more than 96% of the global patent information. Clarivate Analytics has more than 60 years of professional service experience, and aims to provide insights into the frontiers of science and technology and accelerate the pace of innovation by providing trusted data and analysis to customers around the world. DII combines the Derwent World Patent Index (DWPI, 1963-present) with the Derwent Patents Citation Index (DPCI, 1973-present) together. DWPI could offer the unique value-added patent information indexed from over 50 patent issuing authorities, and DPCI could offer the patent citations. DII could provide fast and accurate retrieval of patent information and patent citation information, enabling researchers to grasp the results of global technological innovation quickly and accurately. The core technologies of AI include DL, computer vision, NLP, data mining, etc., and AI algorithm models consist of DT, RF, logistic regression, SVM, NB, neural network, markov, etc. So, the retrieval topics of AI contained all these related technology topics, such as machine learning, deep learning, neural network, algorithm, hidden markov model, random forest, support vector machine, natural language processing, with about 43 search terms related to AI. The search strategy was based on the subject terms of “AI” and “pathology” in DII, which were retrieved in title and abstract. Then, 1941 patents were obtained. The 1941 patents were manually interpreted, and 237 unrelated patents such as compounds and peptides were removed. Finally, 1704 patents were screened. Each patent record in the DII database represents a patent family, which contains one or more patents, with the same technology. So duplicate patents have been combined into one record, and each record represents a technology. The following patent analysis was based on these 1704 patents.

### 2.3 Methods

**Fig 1:**
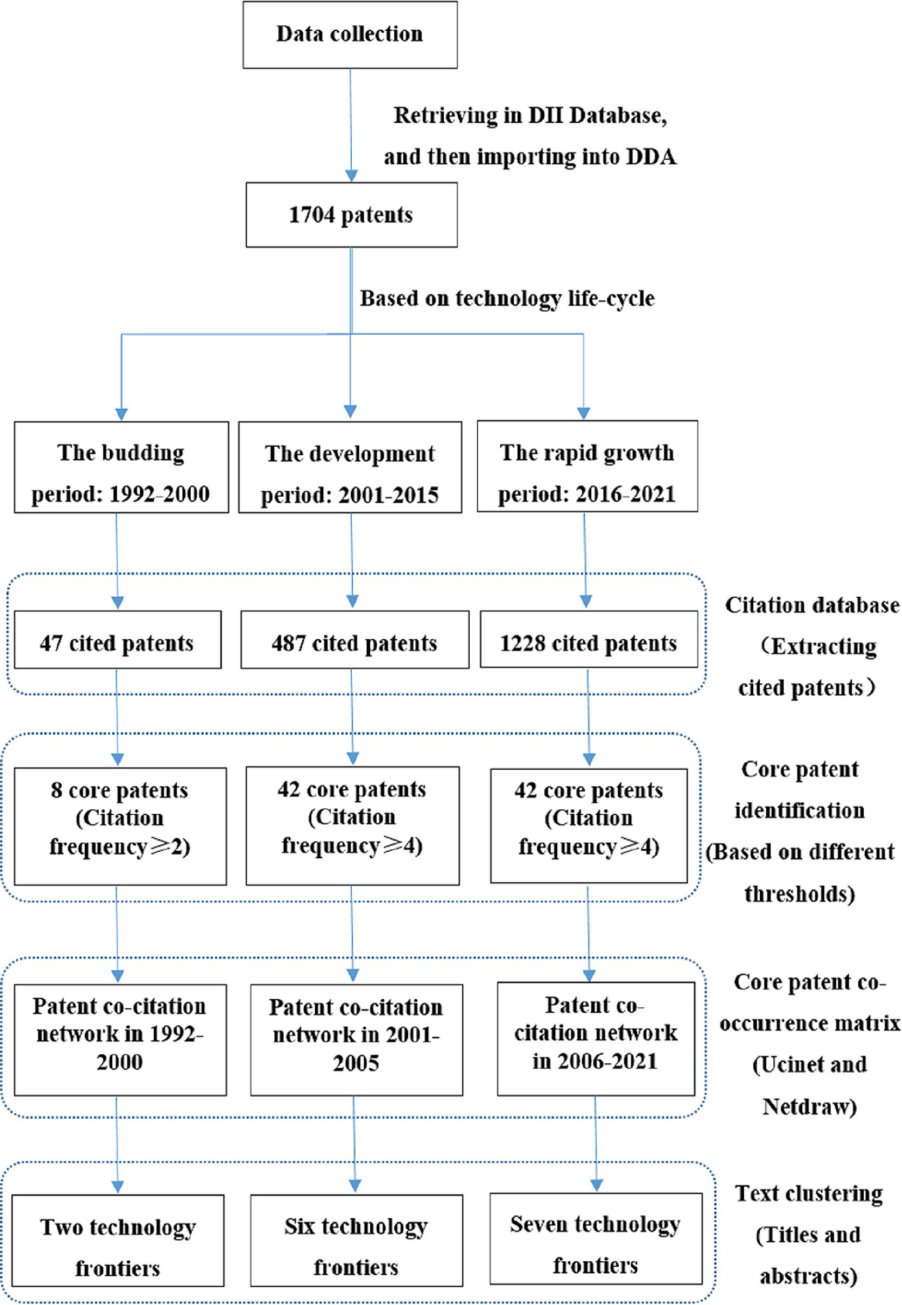
The whole analysis procedure.

The patent data were imported into Derwent Data Analyzer (DDA, Clarivate Derwent, New York, NY, USA) for authority control, and imported into the freely available computer program Ucinet 6 for drawing the patent citation network. Then, the method of text clustering was used to identify the technology frontiers based on the patent citation network. The technology frontiers were initially identified through text clustering, and pathology experts were consulted to finally determine the technology frontiers in each period. The whole analysis procedure is shown in Fig 1.

Patents are important outputs, which could reflect the situation of applied research and other aspects in one field, and also reveal the level of technology and international competitiveness of one country to a certain extent [21–23]. Patent analysis is employed to investigate the research activity and to reveal the current characteristics and status in the field of AI-assisted pathology. The method of text clustering was used to identify the technology frontiers based on the patent citation network, which was according to co-word analysis of the title and abstract of the patents in this field. Patent analysis is a primary method in information science, which utilized quantitative analysis to reveal the development situation in a given field on the basis of patents, which could provide a macroscopic overview of the given field for the researchers, and have been used in many scientific fields, e.g. oncology [11], neurosciences [24] and vaccines [25]. Through the patent analysis of AI-assisted pathology, we could generally understand the main research direction and its development trend.

#### 2.3.1 Patent citation network

The citation relationship between patents could highlight the scientific basis of technology, and point out the inheritance and accumulation relationship between technologies [18, 26–28]. Therefore, the patent citation network according to the citation relationship could describe the technology development context of a specific field [19]. This study extracted patent citations from the collected patent data, selected highly cited patents to form a co-occurrence matrix, and built a patent citation network based on the co-occurrence matrix in each period.

#### 2.3.2 Social network analysis (SNA)

SNA is a structural analysis method that comprehensively uses graph theory and mathematical models to study the relationship between actors and actors, actors and their social networks, and the relationship between one social network and another social network [29–32]. In SNA research, the network structure could be evaluated and examined by analyzing the centrality. Centrality refers to the position of each node in the network [33]. From the difference of statistical calculation methods, centrality could be divided into three types: degree centrality, betweenness centrality, and closeness centrality. The SNA indicators, such as degree centrality, betweenness centrality and closeness centrality, have been used to measure the important or prominent nodes in a network, which could be effective in discovering the central nodes of the network [34, 35]. This study identified patents with high centrality from patent citation network in each period.

*2*.*3*.*2*.*1 Degree centrality*. Degree centrality is the most direct measure to describe node centrality in SNA. The more connections a node has, the more important it is. In a social network, if many individuals have a large number of direct connections with a particular individual, then the individual is at the center of the network [32]. Degree centrality is often used to determine who is dominant in the network. Eq (1) for calculating the degree centrality is as follows:

$${DC}_{i}=\frac{k_{i}}{N-1}$$ (1)

where *DC*_*i*_ indicates the degree centrality. *k*_*i*_ indicates the number of edges that node *i* is directly connected to other nodes. *N* is the number of nodes. *N−1* indicates the number of edges where node *i* is connected to other nodes.

*2*.*3*.*2*.*2 Betweenness centrality*. Betweenness centrality indicates the number of shortest paths through nodes in a network. If an individual is located on multiple shortest paths to other individuals, then the individual is a core individual and has greater betweenness centrality [32]. Eq (2) for calculating the betweenness centrality is as follows:

$${BC}_{i}=\frac{1}{(N-1)(N-2)/2}\sum_{s\neq i\neq t}\frac{n_{st}^{i}}{g_{st}}$$ (2)

where *BC*_*i*_ indicates the betweenness centrality. $n_{st}^{i}$ indicates the number of shortest paths from *s* to *t* through node *i*. *g*_*st*_ indicates the number of shortest paths connecting *s* and *t*. *N* is the number of nodes.

*2*.*3*.*2*.*3 Closeness centrality*. Closeness centrality indicates the shortest distance from a node to other nodes. If the shortest distance from a node to other nodes in the graph is short, then its closeness centrality is high. Closeness centrality is closer to geometric centrality than betweenness centrality [32]. Eq (3) and Eq (4) for calculating the closeness centrality are as follows:

$$d_{i}=\frac{1}{N-1}\sum_{j-1}^{N}d_{ij}$$ (3)

$${CC}_{i}=\frac{1}{d_{i}}$$ (4)

where *CC*_*i*_ indicates the closeness centrality. *d*_*i*_ indicates the average distance from node *i* to other points. *N* is the number of nodes.

#### 2.3.3 Text clustering

Text clustering is a significant method in text mining, and also portion of data mining [36]. Text clustering is an unsupervised learning method, similar documents being grouped into clusters, in order to create clusters that are coherent internally but different from each other [37]. The similarity between documents are determined by calculating the distance between them, and the two documents with the closest distance will be combined. The closer the distance, the higher the similarity [38]. This process is iterated repeatedly. Text clustering has been used extensively in the identification of research fronts in many fields, and also in the evaluation of new technology opportunities [39–41]. In this research, the co-word analysis was used on patent titles and abstracts in the patent citation network, and high-frequency words were selected in the text for cluster analysis, to reflect the technology frontier based on clustering high-frequency words. The Euclidean distance is used to calculate the distance between two points [42, 43], that is, the distance between high-frequency words. Eq (5) for calculating the distance between two points is as follows:

$$(A,B)=\sqrt{{\left(x_{1}-x_{2}\right)}^{2}+{\left(y_{1}-y_{2}\right)}^{2}}$$ (5)

where *A*(*x*_1_, *y*_1_), *B*(*x*_2_, *y*_2_) are any two high-frequency words in space, and (*A*, *B*) is the distance between the two high-frequency words.

## 3. Results

### 3.1 General analysis

**Fig 2:**
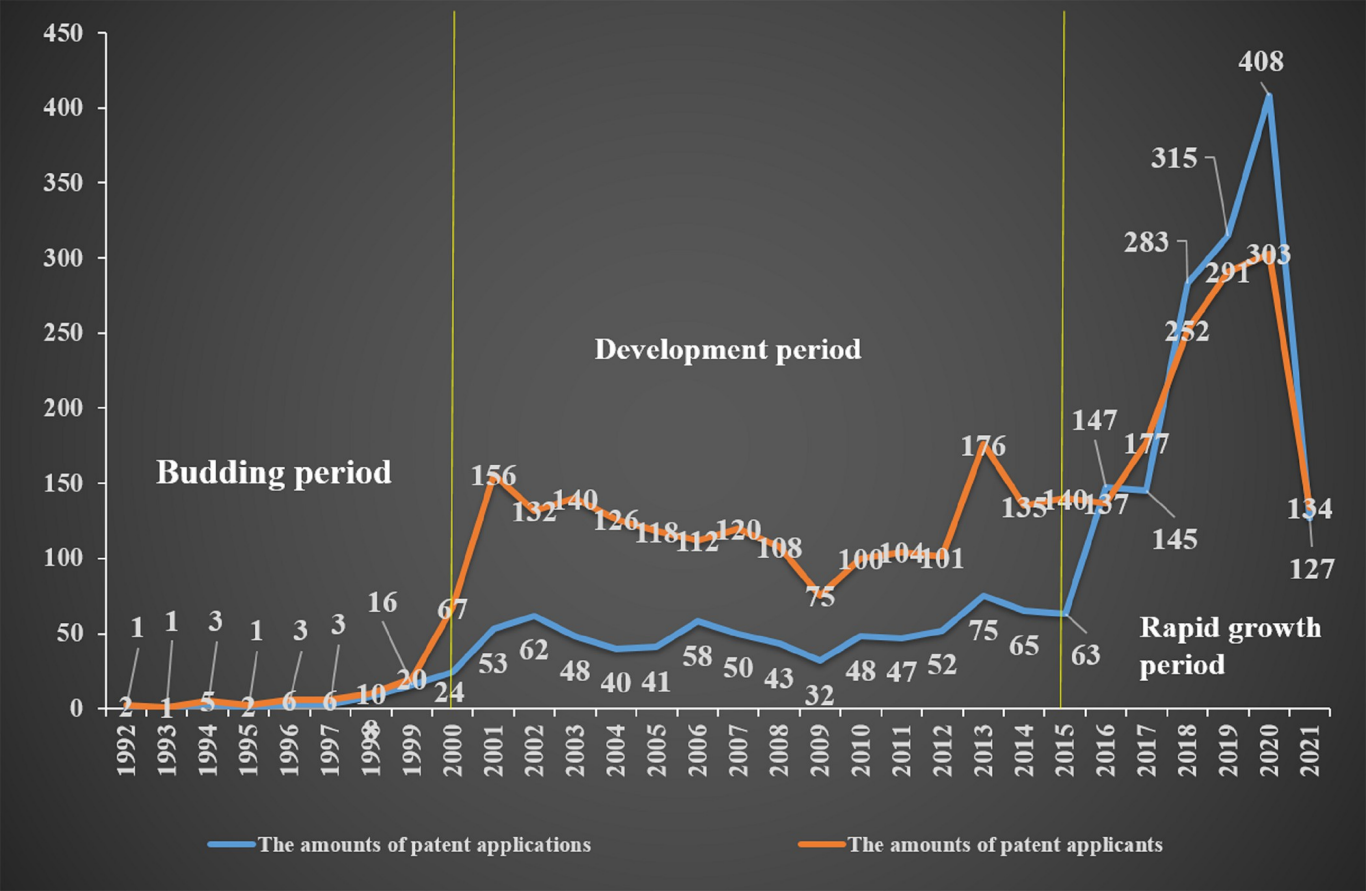
The technology life-cycle of the field of AI-assisted pathology, with the amounts of patent applications and patent applicants. The three stages were (1) Budding period: 1992–2000; (2) Development period: 2001–2015; (3) Rapid growth period: 2016–2021.

Patents of AI-assisted pathology were collected from DII, and 1704 patents were obtained. The first patent application in this field was in 1992 (JP6094706A), which was related to pathological image inspection support device, comprising a device to detect lesions of the tissue and cells of subject. The advantage was that it could supply information of supporting pathological morphogenetic inspection based on pathological images through optical microscope. Then, the number of patent application increased year by year, especially in recent years, showing an obvious increase from 2015. Fig 2 shows the growth of patent applications in the field of AI-assisted pathology. The amounts of patent applications in 2021 did not represent the final trend due to the lag period (eighteen months) from patent application to publication. The amounts of patent application annually increased from 1 in 1992 to 408 in 2020, and showed an obvious increase from 2016, with an increase of 29.07% from 2016 to 2020, indicating that more and more attention had been paid on in recent years. A polynomial regression was done based on the data from 2011 (the year in which patent application began to increase continuously) through 2020. The equation describing the data was y = 6.197x^2^–29.052x + 81.2 with a coefficient of determination R^2^ = 0.9693. If the growth of patent application continued at the same rate, the equation forecasted that there would be 511 patent applications in 2021 and 1040 in 2025, respectively.

### 3.2 Technology life-cycle

This article analyzed the patent citation networks in different periods of AI-assisted pathology to determine the changes of technology frontiers over time. The development period of AI-assisted pathology was divided into three stages (as shown in Fig 2) according to the theory of technology life-cycle [44, 45]. The three stages were as follows:

#### 3.2.1 The budding period: 1992–2000

In budding period (1992–2000), there were only 47 patent applications, and the amounts of patent applications and patent applicants increased slowly, both of which were below 10 in the most of time. During this period, the field of AI-assisted pathology had just begun to develop, with great technology uncertainties, and the technology development was not mature enough.

#### 3.2.2 The development period: 2001–2015

In the development period (2001–2015), there were 487 patent applications, and the amounts of patent applications and patent applicants increased substantially, with the amounts of patent applications being around 60 each year. The trend of changes in the amounts of patent applicants was consistent with that of patent applications, and the amounts of patent applicants (being well over 100 almost every year) far exceeded those of patent applications, indicating that the technology development had attracted a lot of attention during this period, and many patent applicants had poured into this field.

#### 3.2.3 The rapid growth period: 2016–2021

In the rapid growth period (2016–2021), there were 1228 patent applications, and the amounts of patent applications and patent applicants grew rapidly. With the increasing in the amounts of patent applications, those of patent applicants were also increasing, and the trend of the both two was still consistent. The increasing in the amounts of patent applications and patent applicants were 29.07% and 21.95% from 2016 to 2020, respectively. The field of AI-assisted pathology had entered a period of rapid development in recent years, technology had developed rapidly, and AI had brought new development directions and opportunities to pathology.

### 3.3 Core patent identification

There were 1704 patents in the field of AI-assisted pathology, with a total of 11,951 cited patents. Patents with higher citation frequency were agued as having a higher level of innovation, and it was the basis of similar patent technology [46–48]. The patents with the cited frequency more than 4 times were chosen as core patents in the period of 2001–2015 and 2016–2021. The amounts of cited patents were low in the period of 1992–2000, so the patents cited more than 2 times were chosen as core patents. Hence, there were 111 core patents totally. The core patents in each period were used to construct the patent citation network, which were 8 ones in budding period (1992–2000), 42 ones in development period (2001–2015), and 61 ones in rapid growth period (2016–2021).

### 3.4 The constructing of patent citation network and the identification of technology frontiers

The core patents of each stage were used to construct a co-occurrence matrix to form a patent citation network, which were imported into Netdraw for visualization. Patent clustering involving two or more patents could be regarded as a technology frontier. The technology frontiers of each cluster were gained by text clustering combing with expert advice. Each node in the network represented a patent, and the bigger the node, the more times it had been cited by other patents. The connection between two nodes illustrated a co-cited relationship between the two patents. The three identified co-citation networks were discussed in chronological order as follows:

#### 3.4.1 The technology frontiers in the budding period (1992–2000)

**Fig 3:**
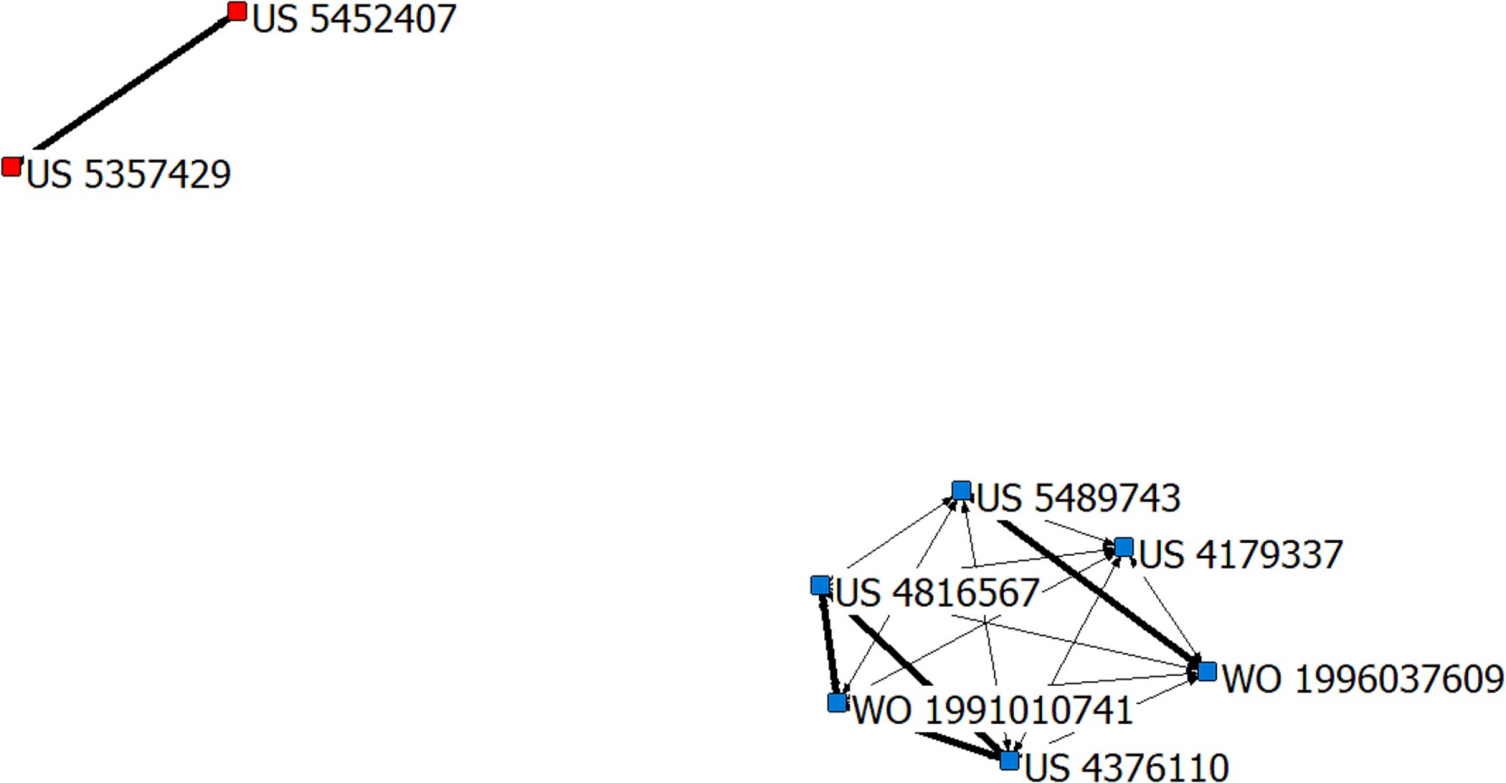
The patent co-citation network of the field of AI-assisted pathology from 1992 to 2000. Note: The subnetwork 1 is with the red nodes, and the subnetwork 2 with the blue nodes.

The co-citation network of the field of AI-assisted pathology from 1992 to 2000 consisted of 8 patents as shown in Fig 3. There were two clusters identified in the budding period (1992–2000). The patents included in each cluster and their technology frontiers are shown in the Table 1. There were two technology frontiers in this period, namely systems and methods for image data processing in computerized tomography (CT), and immunohistochemistry (IHC).

**Table 1:** The technology frontiers from 1992 to 2000.

Subnetwork	Amounts of Patents	Patent Publication Number	Technology Frontiers
**1**	2	US5357429 US5452407	**•** Systems and methods for image data processing in computerized tomography (CT)
**2**	6	US4179337 US4376110 US4816567 US5489743 WO1991010741 WO1996037609	**•** Immunohistochemistry (IHC)

The first cluster included 2 patents (with the red nodes), US5357429 (Title-DWPI: Three-dimensional model generation for computerised tomography) and US5452407 (Title-DWPI: Image data conversion to vector data using CT scanning systems to generate point data and surface tracking to allow B spline polygon to be generated and passed to CAD system), which are related to “systems and methods for image data processing in CT”. The patent US5357429 is related to CT, to generate three-dimensional model using multiple angle tomographic scan planes. It was applied in 1992, with 14 citing patents in DII. The patent US5452407 is related to signal processing, more particularly to a method for converting image data to vector data. It was applied in 1993, with 265 citing patents and 4 patent family members (EP574099A2, CA2087514A1, EP574099A3, US5452407A) in DII. The highly cited patents could be considered as the core patents in a certain technical field. In general, the more times a patent is cited by subsequent patents, the greater the impact on subsequent technology development, and it is more possible in the core position. Therefore, the patent US5452407 was a prominent and critical patent, which was vital for the subsequent technology development and dissemination. There is another highly cited patent WO9507509 (Title-DWPI: Model stereo-lithographic construction), applied in 1994, with 183 citing patents and 7 patent family members (WO9507509-A1, AU9476482-A, EP722588-A1, AU684546-B, US5741215-A, EP722588-B1, DE69432023-E) in DII. This patent uses CT scan data to provide prostheses and anatomical pathology models, and uses a predetermined algorithm to generate a three-dimensional coordinate data set for the pathology. The patent WO9507509 was also a core patent in this field, which was an indispensable part in the knowledge flow of technology development.

The second cluster included 6 patents (with the blue nodes), which was related to “IHC”. There were two highly cited patents pertaining to“IHC”, WO200031534 and WO200139722. The patent WO200031534 (Title-DWPI: In situ analysis of a biological sample comprises using immunohistochemical stains, histological stains and/or DNA ploidy stains together with spectral imaging techniques) was applied in 1999, with 117 citing patents and 9 patent family members (WO200031534-A1, AU200020247-A, US6165734-A, EP1131631-A1, JP2002530676-W, IL142567-A, EP1131631-B1, DE69940270-E, EP1131631-A4) in DII, which had an important impact on the subsequent technology development. This patent is useful for the pathological analysis of cells, and could enable high resolution as well as spectral resolution, which is not possible using prior art imaging methods. The patent WO200139722 (Title-DWPI: Novel DNA encoding immunoregulatory molecule B7-H1, is useful for co-stimulating a T cell for augmenting immunoregulation and for controlling pathologic cell mediated conditions) was applied in 2000, with 115 citing patents and 25 patent family members (AU784634-B2, EP1234031-B2, ES2629683-T3, US9062112-B2, WO200139722-A3, etc.) in DII, which could identify a compound that inhibits an immune response by providing a test compound. IHC is a commonly used technique and method in pathological diagnosis, with higher accuracy, sensitivity, specificity, and low missed diagnosis rate, especially having a huge impact on tumor diagnosis, tumor classification, and prognosis [49, 50]. Many indicators in IHC needs to be accurately and quantitatively analyzed, the results of which are directly related to the targeted therapy and immunotherapy of tumors. IHC suffers from variable consistency, poor reproducibility, quality assurance disparities, and the lack of standardization resulting in poor concordance, validation, and verification [51]. If AI is used to assist the interpretation of IHC results, those problems could be avoided. AI could improve the reliability of interpretation results, and also improve the quality of pathological diagnosis.

#### 3.4.2 The technology frontiers in the development period (2001–2015)

**Fig 4:**
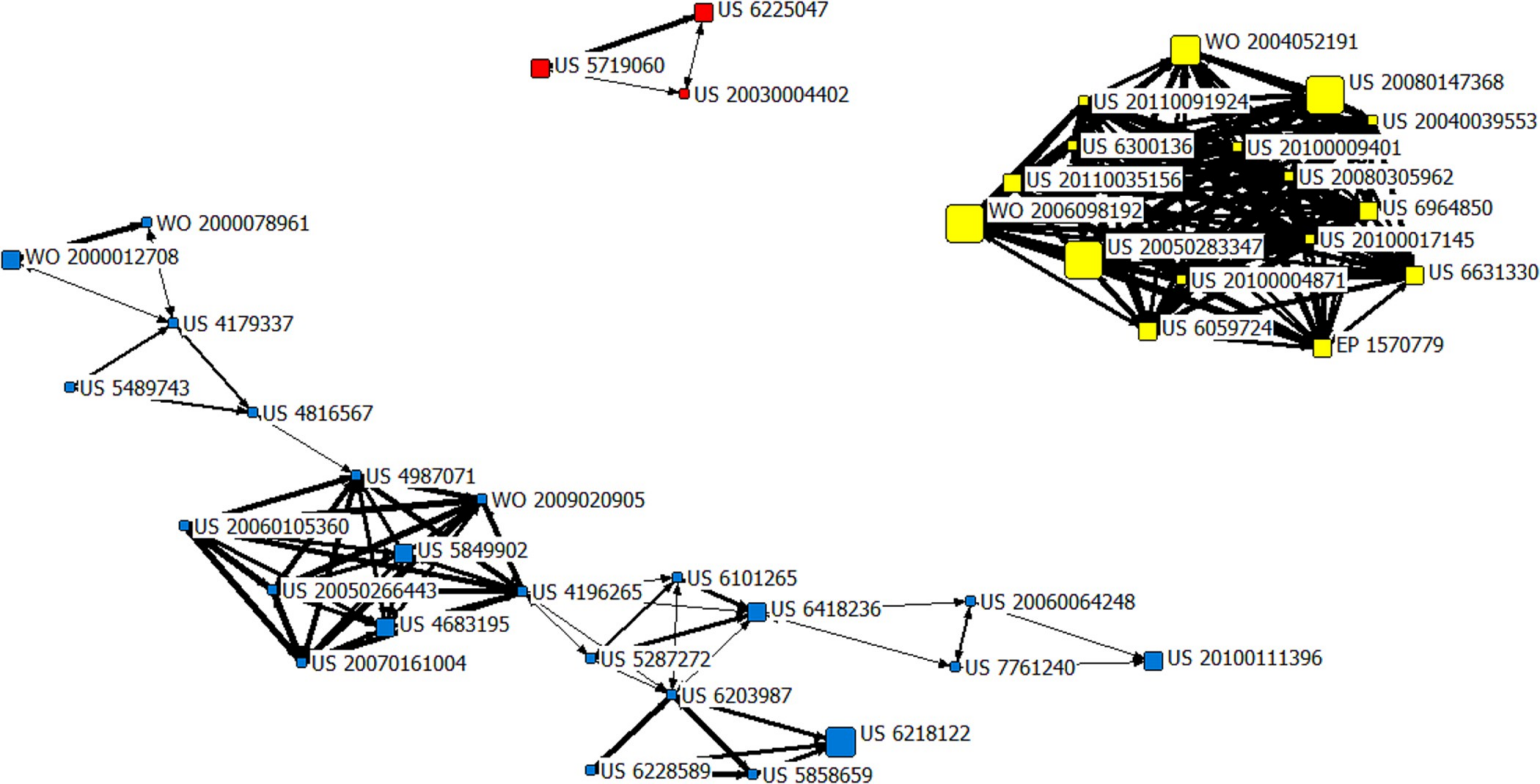
The patent co-citation network of the field of AI-assisted pathology from 2001 to 2015. Note: The subnetwork 1 is with the red nodes, the subnetwork 2 with the yellow nodes, and the subnetwork 3 with the blue nodes.

The co-citation network of the field of AI-assisted pathology from 2001 to 2015 consisted of 42 patents as shown in Fig 4. There were three clusters identified in the development period (2001–2015). The patents included in each cluster and their technology frontiers are shown in the Table 2. There were six technology frontiers in this period, namely spectral analysis methods of biomacromolecules, pathological information system, diagnostic biomarkers, molecular pathology diagnosis, and pathological diagnosis antibody.

**Table 2:** The technology frontiers from 2001 to 2015.

Subnetwork	Amounts of Patents	Patent Publication Number	Technology Frontiers
**1**	3	US5719060 US6225047 US20030004402	**•** Spectral analysis methods of biomacromolecules
**2**	16	US20050283347 US20080147368 WO2006098192 WO2004052191 EP1570779 US20110035156 US6059724 US6631330 US6964850 US20040039553 US20080305962 US20100004871 US20100009401 US20100017145 US20110091924 US6300136	**•** Pathological information system **•** Diagnostic biomarkers
**3**	23	US6218122 US20100111396 US4683195 US5849902 US6418236 WO2000012708 US20050266443 US20060064248 US20060105360 US20070161004 US4179337 US4196265 US4816567 US4987071 US5287272 US5489743 US5858659 US6101265 US6203987 US6228589 US7761240 WO2000078961 WO2009020905	**•** Molecular pathology diagnosis **•** Pathological diagnosis antibody

The first cluster included 3 patents (with the red nodes), US5719060 (Title-DWPI: Sample desorption in mass spectrometry), US6225047 (Title-DWPI: Identification of analytes that are present in first and second biological samples, includes exposing samples to different selectivity conditions, and detecting analytes in first and second samples by desorption spectrometry) and US20030004402 (Title-DWPI: Classifying a biological state from biological data useful for the diagnosis of disease e.g. cancer comprises detecting a discriminatory pattern and applying the pattern to classify known and unknown data samples), which are related to “spectral analysis methods of biomacromolecules”. For analysis of biomacromolecules, a sensitive, specified and reliable method is indispensable. The patent US5719060 is regarding the field of mass spectrometry (MS), providing methods and apparatus for desorption and ionization of analytes. MS is a technique that could elucidate the molecular structure and molecular weight of analytes [52]. The patent US6225047 is pertaining to the methods of identifying analytes that are differentially present between two samples, which uses retentate chromatography to generate difference maps. The patent US20030004402 is concerning a process for determining a biological state through the discovery and analysis of biological data patterns. The detection of changes in biological states, particularly the early detection of diseases, has been a central focus of the medical research and clinical community [53, 54].

The second cluster included 16 patents (with the yellow nodes), which was related to two technology frontiers, involving “pathological information system” and “diagnostic biomarkers”. There were several important patents related to “pathological information system”, which could implement the extraction, classification and evaluation of pathological information, such as US2002039434, US2012087556, WO2008005426 and so on. The patent US2002039434 (Title-DWPI: Computerized medical decision support system uses computer program product that provides data derived from examination of digital images of tissue specimen according to criteria for histopathological analysis) was applied in 2001, with 16 citing patents and 2 patent family members (US2002039434-A1, US7027627-B2) in DII. It provides a computer system, which is used for examining digital images of tissue specimen, analyzing and diagnosing benign and malignant pathologies. The patent US2012087556 (Title-DWPI: Computer-implemented feature extraction method for classifying pixels of digitized pathology image involves classifying by system unit or all of digitized pathology image based on determined first region, and determined subsequent regions) was applied in 2010, with 13 citing patents and 2 patent family members (US2012087556-A1, US8351676-B2) in DII. This patent is used for a feature extraction method for classifying pixels of digitized pathology image. The patent WO2008005426 (Title-DWPI: Internet based, patient-specific data evaluation system for use in medical prognoses enables different branches of medicine to be integrated to improve the quality of a prognosis) was applied in 2002, with 10 citing patents and 7 patent family members (WO2003021478-A2, DE10143712-A1, EP1423806-A2, AU2002333775-A1, US2004236723-A1, JP2005501625-W, WO2003021478-A3) in DII. It offers a data evaluation system, which is used to be integrated to improve the quality of a prognosis by integrating clinical, pathological and molecular biological data. The goal of pathological information system is to develop a comprehensive platform that could be utilized across contexts (e.g., basic research laboratories, brain banks, and clinical pathology laboratories) with variability in sampling protocols, tissue section quality, staining methodology, and pathological features, which requires the creation of computational infrastructure and large pathological datasets containing richly varied high-quality annotations. Based on the integrated multi-element pathological information system, it is able to apply trained networks for larger disease-specific cohorts and to generate quantitative data for clinicopathological correlations, as well as for molecular and genetic studies, and enables further diagnostic and therapeutic strategies [55].

Another technology frontier in the second cluster was “diagnostic biomarkers”. Biomarkers are widely used for diagnosis, efficacy assessment and prognosis prediction in clinical, and related patent applications are very active. Diagnostic Biomarkers are used to detect or confirm disease status, or identify different disease subtypes. For example, the patent WO2003019193 (Title-DWPI: Aiding kidney disease diagnosis comprises detecting a protein marker and correlating it with a probable diagnosis of a kidney disease) was applied in 2002, with 17 citing patents and 4 patent family members (WO2003019193-A1, AU2002332832-A1, US2005260678-A1, US7297556-B2) in DII. It offers methods which could determine the patient’s pathological status by using of minute quantities of crude samples. Also, it includes an AI algorithm of determining closeness-of-fit between the computer-readable data and diagnosis data. The patent US2011091081 (Title-DWPI: Acquiring data for analyzing expression of biomarkers in cells in their tissue of origin comprises e.g. acquiring image of tissue specimen, segmenting images into individual cells, and determining expression level of biomarkers within cells) was applied in 2009, with 79 citing patents in DII. This patent could acquire data and analyze patterns of expression of multiple biomarkers. With the development of immunology, molecular biology and genomics, discovering reliable early diagnostic biomarkers had become a research hotspot. Diagnostic biomarkers are one of the important basis for clinical disease diagnosis, and are usually used as the inclusion criteria for specific subjects in clinical trials [56–59]. Protein biomarkers play an essential role in diagnosis of cardiovascular disease [60]. The exponential increase in the use of molecular biomarkers as diagnostic, prognostic, and predictive aids in the management of cancer patients highlighted the increasing importance of molecular biology in oncology [61]. Biomarkers have enabled new clinical and pathological insights into the mechanisms underlying all kinds of tumor and also have facilitated improvements in the diagnostic workup, sub-classification, outcome stratification, and personalized therapy for tumor patients [62].

The third cluster included 23 patents (with the blue nodes), which was also related to two technology frontiers, involving “molecular pathology diagnosis” and “pathological diagnosis antibody”. Molecular diagnostics provides a powerful method to detect and diagnose various diseases. The patent applications of “molecular pathology diagnosis” were active, which had laid an important technological foundation for subsequent technology diffusion and dissemination. The patents with important influence were as follows. The patent WO2008008430 (Title-DWPI: Diagnosing colon cancer-related disease comprises measuring the level of miR gene product in a test sample from the subject) was applied in 2007, with 54 citing patents and 68 patent family members (WO2008008430-A3, EP2041317-A2, AU2007272947-A1, JP2009543552-W, CN101657547-A, US2010257618-A1, etc.) in DII. This patent provides a method of testing for initiation, predisposition, or decreased survival prognosis of a disease response, which could indicate the developing risk of the colon cancer-related disease, by measuring the level of miR gene. The patent WO2010129934 (Title-DWPI: Diagnosing thyroid disease in subject involves providing DNA sample from subject, detecting presence of specific polymorphism, and determining whether the subject has malignant or benign thyroid condition) was applied in 2010, with 24 citing patents and 21 patent family members (WO2010129934-A3, EP2427575-A2, CN102459636-A, US2012220474-A1, JP2012525855-W, HK1165837-A0, etc.) in DII. This patent provides a method of diagnosing cancer based on a DNA sample, with the specificity or sensitivity greater than 70%. And also, it includes algorithms to assign a statistical probability to the accuracy of the diagnosis of genetic disorders. The confirmation of such diagnosis allows early detection and subsequent medical counseling that helps specific patients to undergo clinically important drug trials [63]. With the advent of precise tumor therapy and targeted therapy drugs, the clinical application of molecular pathology diagnosis has developed rapidly and become increasingly popular. Using molecular biology techniques to detect and determine whether there is a mutation in the molecular genetics of tumor tissue, thereby guiding accurate clinical diagnosis and treatment, and striving for more benefits for patients, this concept has been accepted by more and more clinicians [62, 64]. Advances in molecular biology and molecular genetics have promoted pathological diagnosis from traditional morphological to molecular level research. By analyzing the genetic abnormalities of tumor patients, including common gene amplification, chromosomal translocation/gene fusion, and gene mutation, it could provide more valuable information for pathological diagnosis and clinical treatment.

Another technology frontier in the third cluster was “pathological diagnosis antibody”. Antibodies could make suitable biomarkers for the prediction of disease because of being relatively easily measured in bodily fluids by a variety of (usually inexpensive) immunoassays. Diagnostic antibodies are widely used in in-vitro diagnostic kits, including enzyme-linked immunosorbent assay (ELISA), chemiluminescence (CL), colloidal gold, turbidimetric inhibition immuno assay, with a wide range of patents. The patent WO2004094460 (Title-DWPI: New purified brain-type natriuretic peptide (BNP) fragment for diagnosing cardiovascular diseases such as stroke, congestive heart failure, cardiac ischemia or hypertension) was applied in 2005, with 59 citing patents and 10 patent family members (WO2004094460-A2, US2005064511-A1, EP1616181-A2, JP2006527190-W, US7341838-B2, US2008160540-A1, EP1616181-B1, DE602004022527-E, WO2004094460-A3, EP1616181-A4) in DII. This patent is used to diagnose diseases with a monoclonal antibody, and contains a variety of technologies, such as immunoassay, training learning algorithm and so on. The patent WO200272786 (Title-DWPI: New transmembrane serine protease 7 (MTSP7) polypeptide for diagnosing neoplastic diseases, monitoring tumor progress or therapeutic effectiveness, or identifying MTSP7 modulators for treating tumors or cancers) was applied in 2002, with 3 citing patents and 6 patent family members (WO200272786-A2, US2003008372-A1, AU2002305052-A1, AU2002305052-A8, US7125703-B2, WO200272786-A3) in DII. This patent is about a new polypeptide, which is useful for detecting or diagnosing a neoplastic disease, a pre-malignant lesion, a malignancy or other pathologic condition. Diagnosis antibodies are now used widely in relation to physical disease, both systemic and central nervous system (CNS)-restricted, to improve clinical understanding and management, reflecting an increasing awareness of the involvement of the immune response in aspects of many diseases [65]. Antibody testing could be used as a diagnostic tool in autonomic disorders, for example, diagnostic evaluation of autonomic disorders could be supplemented by testing for paraneoplastic antibodies and antibodies against membrane receptors [66]. Research had found that antinuclear antibody (ANA) may be more common in schizophrenia than in controls [67]. In addition, a positive test result for ANAs may indicate the presence of a systemic autoimmune disease, such as systemic lupus erythematosus (SLE), drug-induced lupus, scleroderma (SS), and rheumatoid arthritis (RA) [68]. Furthermore, last year, Diadem, an Italian diagnostic reagent company, announced that its AD early diagnosis antibody could advance AD diagnosis by 6 years. The technologies had patent applications in many countries and regions, and widely deployed around the world. The first patent is IT1426278 (Title-DWPI: Anti-human p53 antibody in kit used for detecting isoform of human p53 protein in sample, and diagnosing AD, is specifically binds to linear epitope of human p53, where the anti-human p53 antibody comprises ten amino acids) which was applied in 2014, with 3 citing patents and 22 patent family members (WO2016050630-A1, IT1426278-B, EP3201234-A1, CN107001453-A, JP2017534595-W, US2018057572-A1, HK1241896-A0, etc.). The anti-human p53 antibody is monoclonal antibody, which is used for detecting isoform of human p53 protein in sample, and diagnosing AD, predisposition affected by mild cognitive impairment and predisposition of cognitive frailty during aging in subject. More innovative drug discovery for AD turns to discover early markers, and early diagnosis of AD is becoming more and more important.

#### 3.4.3 The technology frontiers in the rapid growth period (2016–2021)

**Fig 5:**
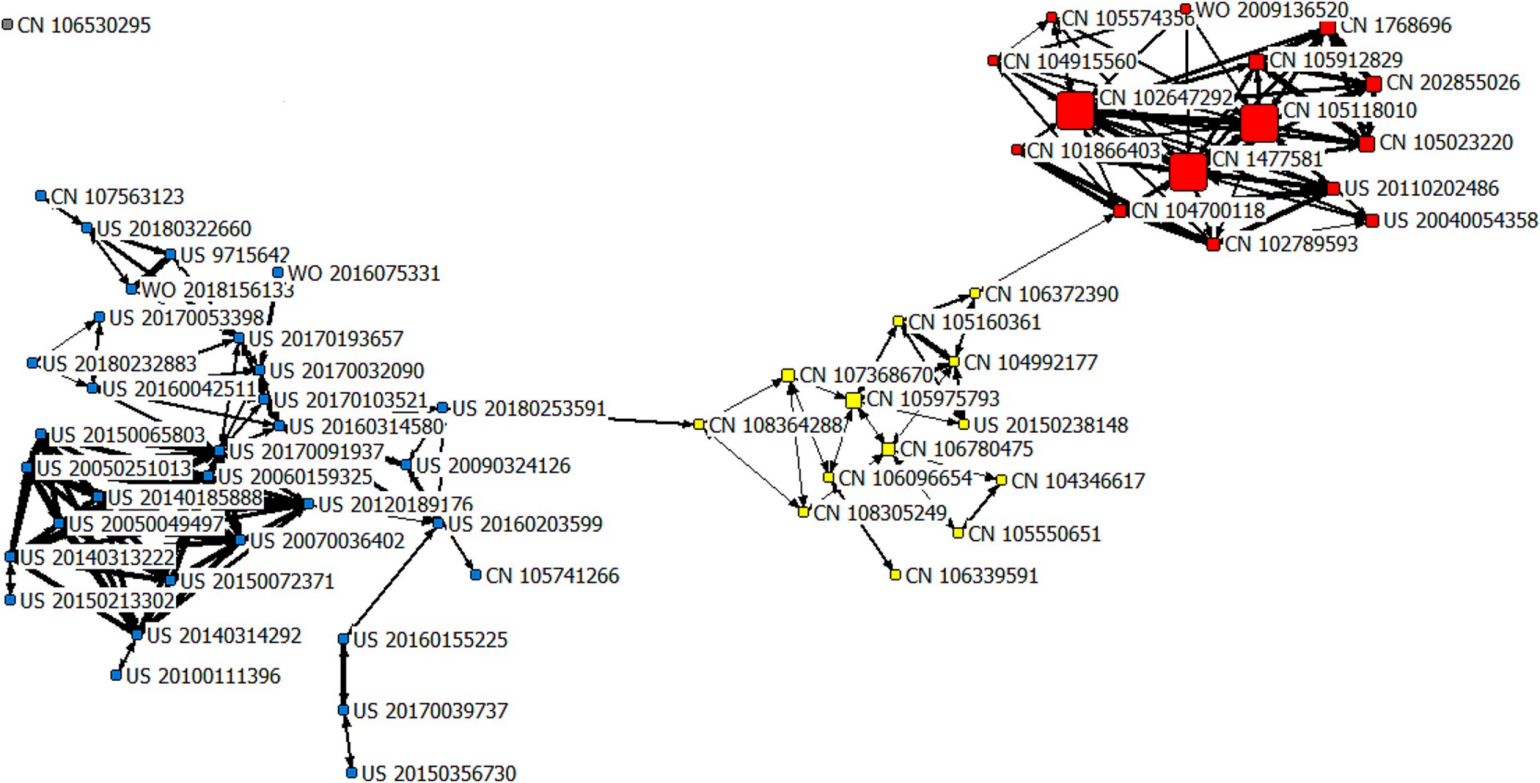
The patent co-citation network of the field of AI-assisted pathology from 2016 to 2021. Note: The subnetwork 1 is with the yellow nodes, the subnetwork 2 with the red nodes, and the subnetwork 3 with the blue nodes.

The co-citation network of the field of AI-assisted pathology from 2016 to 2021 consists of 61 patents as shown in Fig 5. There were three clusters identified in the rapid growth period (2016–2021). The patents included in each cluster and their technology frontiers are shown in the Table 3. There was one patent CN106530295 had no connection with other patents, which was not in any citation network, so it was not included in the analysis. There were six technology frontiers in this period, namely DP, DL algorithms—CNN, disease prediction models, computational pathology, pathological image analysis method, and intelligent pathological system. The first cluster included 12 patents (with the yellow nodes), which were related to two technology frontiers, involving “DP” and “DL algorithms—CNN”. With the growth of computing power, AI has expanded the scope of DP, evolving from the initial digital process to digital image detection, segmentation, diagnosis and analysis methods. DP is to scan and collect high-resolution digital images through an automatic microscope or optical magnification system, and then apply computers to automatically perform high-precision multi-view seamless stitching and processing, in order to obtain high-quality visualization data for application [69]. The core content of DP is to electronically digitize and network traditional pathological images. China had active patent applications in “DP”, especially related to full slice DP image (e.g., CN107665492, CN108846828, CN110335668, and CN106408573). The patent CN107665492 (Title-DWPI: Colorectal panoramic digital pathological image tissue segmentation method based on deep network comprises obtaining a panoramic DP image of the colorectal under magnifying glass) was applied in 2017, with 6 citing patents in DII. It provides a method for colorectal panoramic digital pathological image tissue segmentation based on deep network, with accurate classification and high classification speed. Some patents were about image processing, such as WO2020243583 and US2020349707, both of which were applied in 2020, with 5 patent family members each. The patent WO2020243583 (Leica Biosystems Imaging Inc.) offers a DP system, with an AI processing module for processing image data from a histological image, which could shorten the time between biopsy and diagnosis, and enable an entire image to be processed. Leica Biosystems Imaging Inc. (Vista, CA, USA), with about 100 patents, many of which are in regard to DP. It is one of major offices of Leica Biosystems (Nussloch, Germany), a cancer diagnostics company, which is committed to delivering improved quality, integrated solutions, and optimized efficiencies leading to breakthrough advances in diagnostic confidence. The patent US2020349707 (Huron Technologies Inc.) develops feature extractor and classifier for the image patches by training a neural network on a DP database, which provides an image diagnostic system related to histological and histopathological images. At present, AI has been used to explore cytological screening, quantitative analysis, histopathological diagnosis and prognosis judgment based on pathological images. With the rapid development of computer and digital imaging equipment, the WSI has rapidly appeared in the medical field, which makes up for the defects of traditional glass slice and also brings a huge workload. In 2017, the US Drug and Food Administration (FDA) cleared the use of the first WSI system for primary diagnostics, the IntelliSite Pathology Solution (PIPS) [3]. Then, in 2019, FDA approved another scanner, Leica Aperio AT2 DX System, for review and interpretation of digital surgical pathology slides prepared from biopsied tissue [70]. AI assisting pathologists in WSI analysis could not only improve the consistency of diagnosis, but also greatly reduce the workload of pathologists. It has become routine in the pathology field to use the WSI to scan the image on the slide into a digital picture, and then use the network transmission to remote consultation and academic exchange of difficult cases [71].

**Table 3:** The technology frontiers from 2016 to 2021.

Subnetwork	Amounts of Patents	Patent Publication Number	Technology Frontiers
**1**	12	CN105975793 CN107368670 CN106780475 CN104992177 CN106339591 CN108305249 CN104346617 CN105160361 CN105550651 CN106096654 CN106372390 US20150238148	**•** Digital pathology **•** Deep learning (DL) algorithms—convolutional neural networks (CNN)
**2**	15	CN1477581 CN102647292 CN105118010 CN105023220 CN105912829 CN1768696 CN202855026 CN104700118 US20110202486 CN102789593 US20040054358 CN105574356 CN101866403 CN104915560 WO2009136520	**•** Disease prediction models
**3**	33	US20120189176 US20180232883 US20180253591 WO2018156133 CN108364288 US20100111396 US20150072371 US20150213302 US20160042511 US20160203599 US9715642 CN105741266 CN107563123 US20050049497 US20050251013 US20060159325 US20070036402 US20090324126 US20140185888 US20140313222 US20140314292 US20150065803 US20150356730 US20160155225 US20160314580 US20170032090 US20170039737 US20170053398 US20170091937 US20170103521 US20170193657 US20180322660 WO2016075331	**•** Computational pathology **•** Pathological image analysis method **•** Intelligent pathological system

“DL algorithms–CNN” was another technology frontier in the first cluster. DL algorithms play a crucial role in determining the features and could handle the large number of processes for the data that might be structured or unstructured. The CNN algorithm is the most widely used [4]. In recent five years, China had applied for a large number of patents on CNN. For example, there were some patents (e.g., CN109086836, CN108509991, and CN108765408) for processing pathological images based on CNN, which were all applied in 2018. The patent CN109086836 is used for cancer pathological image automatic discriminating, the patent CN108509991 is used for liver pathology image classifying, and the patent CN108765408 is used for constructing cancer pathology images of virtual case library. Also, there were some patents (e.g., CN109003659, CN107368670, and CN107368671) were in connection with pathological diagnosis support system, which all had CNN model training unit, and these systems could improve the accuracy and working efficiency. The three patents were applied in 2017 and 2018, respectively, and the patent assignees was all Shenzhen Imsight Medical Technology Co Ltd (Shenzhen, China). In addition to China, other countries also had corresponding patent layouts in this technology frontier. The patent US2020349707 (Title-DWPI: Image diagnostic system for identifying tissue types in image patch according to hierarchical histological taxonomy, has processor to develop convolutional neural network (CNN) based on training image patches in digital pathology database) was applied in 2020, with 5 patent family members (US2020349707-A1, WO2020223798-A1, CA3138959-A1, EP3963508-A1, US11276172-B2) in DII. This patent is also concerning image diagnostic system, which develops a CNN processor based on the set of training image patches. Its advantages are to reduce cognitive workload required of pathologists by narrowing the visual search area and highlighting regions of diagnostic relevance, thus enabling pathologists to focus on diagnosing relevant regions of interest. CNN is an efficient recognition method developed in recent years, which could effectively reduce the dimension of pictures with a large amount of data without affecting the results, and retain the characteristics of the pictures more completely. The trained CNN model could obtain the characteristics of pathological slices comprehensively and accurately without being restricted by professional factors, which is suitable for WSI analysis of big data, and its application scope includes tumor histopathological classification, lymph node metastasis cell detection, and prediction of prognosis in cancer patients, etc [69, 72].

The second cluster included 15 patents (with the red nodes), which was related to only one technology frontier, that is, “disease prediction models”. This technology frontier had grown tremendously with the evolving of DL techniques. DL techniques require large amounts of data in which computer algorithms could find fixed patterns. These fixed patterns contribute to the formation of algorithmic models, which could be used to predict the occurrence of events. China had applied for a large number of patents in“disease prediction models”, and there were more than 40 patents related to neural network model based predicting disease method. The patent CN113782221 (Title-DWPI: Self-training learning based disease prediction device for use in artificial intelligence technology field, has input module for inputting patient pathological data into target disease prediction model to obtain target disease prediction result) was applied by Ping An Technology (Shenzhen, China) in 2021. This patent could achieve self-training learning based disease prediction, which reduces manpower and material resources and data deficiency for model training to obtain accurate robust prediction model. On the basis of big data, combined with DL and pathological images, disease prediction models could be constructed. The GOOGLE LLC (Mountain View, CA, USA) applied a patent WO2021021329 (Title-DWPI: Medical image interpretation, e.g., for mammograms, involves feeding cropped pairs of images into deep learning model trained to make prediction of probability of disease state and generating prediction for each pair) in 2020, with 1 citing patent and 2 patent family members (WO2021021329-A1, EP3973539-A1) in DII. This invention is based on the cropped images, which are fed into a DL model, and trained for making a prediction of disease probability. The accurate diagnosis of tumors is very important for the selection of treatment options and prognosis of patients. Pathological diagnosis is considered to be the “gold standard” for tumor diagnosis. There was a research which developed and validated a diagnostic nomogram model with imaging features to predict preoperative pathological grade in meningioma patients [73]. Also, another research developed an explainable model for predicting pathological diagnosis and survival in patients with interstitial lung disease [74]. The breakthrough progress of WSI and DL provides new development opportunities for computer-aided diagnosis and prognosis prediction.

The third cluster included 33 patents (with the blue nodes), which was also related to three technology frontiers, involving “computational pathology”, “pathological image analysis method” and “intelligent pathological system”. The greatest advantage of computational pathology is reducing errors of diagnostic and classification. The accuracy of computational pathology applications largely depends on a large amount of data, reliable hardware and software, and the support of the network environment. Some patents of computational pathology were involving quantitative imaging analysis, and there were two related US patents with more than 10 citing patents. The patent US2017046839 and US2019244347 both disclose a hierarchical analytics framework, which advantageously improve the trainability and resulting reliability of the algorithms. The patent US2017046839 (Title-DWPI: System for analyzing pathology utilizing quantitative imaging, has non-transient storage medium including processor implementing analyzer module configured to utilize second set of algorithms to identify and characterize medical conditions) was applied in 2016, with 21 citing patents and 3 patent family members (US2017046839-A1, WO2017096407-A1, US10176408-B2) in DII. This patent uses the radiological imaging to provide surrogate measures for predicting clinical outcome. The patent US2019244347 (Title-DWPI: Method for computer aided phenotyping of pathology using enriched radiological dataset for quantitative imaging of anatomical region of patient, involves using machine learned classification approach to determine phenotype for pathology) was applied in 2018, with 15 citing patents and 2 patent family members (US2019244347-A1, US11094058-B2) in DII. This patent utilizes the unsupervised learning application, and it improves the efficiency in DL approaches based on the histology annotated dataset. Computational pathology not only facilitates more efficient pathology workflows, but also provides a more comprehensive and personalized view to help pathologists solve complex diseases and improve patient diagnosis and treatment progress [75, 76]. Computational pathology has the potential to change the core functions of traditional pathology.

Breakthroughs in DL methods improves pathological image analysis method. Pathological image analysis based on DL includes classification, detection, segmentation, registration, retrieval, etc. There was one Chinese patent CN107680088 (Title-DWPI: Method for analyzing medical image, involves obtaining medical image data, obtaining multi-scale training sample data by using depth neural network model according to consistency rule data and output result) was applied in 2017, with 22 citing patents and 3 patent family members (CN107680088-A, US2019102878-A1, US10810735-B2) in DII. The image analysis method in this patent enables accelerating training process by adopting the deep neural network (DNN) model, and improves the accuracy of auxiliary diagnosis decision. There were two EP patents, EP3486836 and EP3531339, in connection with analyzing image using DL algorithm of neural network structure. The patent EP3486836 (Title-DWPI: Method for analyzing image of tissue or cell using deep learning algorithm of neural network structure, involves generating data indicating region of cell nucleus in analysis target image by deep learning algorithm) was applied in 2018, with 2 citing patents and 6 patent family members (EP3486836-A1, US2019156481-A1, JP2019095853-A, CN109871735-A, US11074692-B2, US2021312627-A1) in DII, which improves the degree of learning of the neural network by repeating the DL processes. The patent EP3531339 (Title-DWPI: Image analysis method for analyzing image of tissue using deep learning algorithm of neural network structure, involves generating data indicating layer structure configuring tissue in analysis target image by deep learning algorithm) was applied in 2019, with 1 citing patent and 6 patent family members (EP3531339-A1, US2019266486-A1, CN110197714-A, JP2019148950-A, US11276000-B2, EP3531339-B1) in DII, which enables the user to obtain the result of the image analysis process without acquiring the window size database and the algorithm database from the DL apparatus. The pathological image analysis task focuses on analyzing the pathological image itself, which provides a good foundation for subsequent pathological-related tasks.

In terms of intelligent pathology, the system takes pathological images as the core, and combines multi-dimensional data, such as genetic data and medical history data, to make judgments, which provides diagnostic results that doctors could refer to, in order to improve the diagnostic accuracy and work efficiency. The high-pace rise in advanced computing and imaging systems has given rise to intelligent pathological system. The technology frontiers of“intelligent pathological system” had developed rapidly in recent years, and there were many patent applications in these five years. There was one patent CN110767312 (Title-DWPI: Artificial intelligence aided pathological diagnosis system, has memory for storing programs, where program includes steps of receiving final analysis report from specialist doctor and outputting final analysis report), applied in 2019, with 1 citing patent in DII. The patent provides a system which could implement a preliminary screening of the DP files, and directly generate a preliminary analysis report to reduce workload of pathological doctors. Intelligent pathological system could also make disease predictions, such as US2017193175 and US2017352157, and patent assignees was both Case Western Reserve University (CWRU). The patent US2017193175 (Title-DWPI: Non-transitory computer-readable storage device for predicting recurrence of non-small cell lung cancer, has set of instructions to control computer aided diagnosis system to classify region of tissue as non-recurrence or recurrence region) was applied in 2016, with 7 citing patents and 2 patent family members (US2017193175-A1, US10049770-B2) in DII. The invention is regarding a computer assisted diagnosis system, which is controlled to generate a personalized treatment plan based on the classification and a radiological image. The patent US2017352157 (Title-DWPI: Non-transitory computer-readable storage device for predicting low infiltrating lymphocyte density, has set of instructions for controlling diagnosis system to generate treatment plan based on classification and radiological image) was applied in 2017, with 2 patent family members (US2017352157-A1, US10346975-B2) in DII. The invention improves existing approaches to predict non-small cell lung cancer (NSCLC) recurrence. DL greatly promotes the development of intelligent pathological system in many field, such as DL-based automatic computer-aided diagnosis system for diabetic retinopathy [77], a DL approach for fast automatic vertebrae detection and localization in pathological CT scans [78]. Moreover, FDA had approved a DL-based autonomous AI diagnostic system to detect diabetic retinopathy on the images of retinal fundus [79].

### 3.5 The high centrality patents in each period

Several centralities of SNA could be used as indicators for judging core technologies, which could be obtained by using the SNA software Ucinet. Then, the degree centrality, betweenness centrality and closeness centrality were chosen. The patent with a higher degree centrality has more direct relations with other patents in the co-citation network, which indicates that it has greater influence and importance than others. The patent with a higher betweenness centrality plays an important role in connecting other patents, which means it is necessary in the evolution and development of technology. The patent with a higher closeness centrality has shorter distances to other patents, which could result in more rapid diffusion of knowledge with other patents in its field. Those patents with highest degree centrality, betweenness centrality and closeness centrality of each period are presented in Table 4.

**Table 4:** The highest centrality patents in each period.

Development stage	Centrality	Patent Publication Number	Patent title
**Budding period (1992–2000)**	Degree Centrality	US4816567	Recombinant immunoglobin preparations
		US4376110	Immunometric assays using monoclonal antibodies
		WO1991010741	Generation of xenogeneic antibodies
**Development period **(**2001–2015)**	Degree Centrality	US20100017145	Method of evaluating cancer state, cancer-evaluating apparatus, cancer-evaluating method, cancer-evaluating system, cancer-evaluating program and recording medium
		US20110091924	Method of evaluating cancer type
		US20080305962	Methods and Kits for the Prediction of Therapeutic Success, Recurrence Free and Overall Survival in Cancer Therapies
		US20100004871	Identities, specificities, and use of twenty two (22) differentially expressed protein biomarkers for blood based diagnosis of breast cancer
		US20100009401	Method of evaluating colorectal cancer, colorectal cancer-evaluating apparatus, colorectal cancer-evaluating method, colorectal cancer-evaluating system, colorectal cancer-evaluating program and recording medium
		US6300136	Methods for diagnosis and treatment of tumors in humans
		US20040039553	Diagnosis method of inflammatory, fibrotic or cancerous disease using biochemical markers
	Between Centrality	US4196265	Method of producing antibodies
	Closeness Centrality		
**Rapid growth period (2016–2021)**	Degree Centrality	US20170091937	Methods and systems for assessing risk of breast cancer recurrence
	Between Centrality	US20180253591	Predicting cancer progression using cell run length features
	Closeness Centrality		

#### 3.5.1 The high centrality patents in the budding period (1992–2000)

In the budding period (1992–2000), there were three patents with the highest degree centrality, that is, US4816567, US4376110 and WO1991010741. The patent US4816567 is about immunoglobin. The patents US4376110 and WO1991010741 are related to monoclonal antibodies. They are all related to IHC. One of the technology frontiers in the budding period was IHC, and immunoglobin and monoclonal antibodies could be used for judging the benign and malignant tumors, and determining tumor staging [80]. The degree centrality also confirmed our findings of technology frontiers.

#### 3.5.2 The high centrality patents in the development period (2001–2015)

In the development period (2001–2015), there were seven patents with the highest degree centrality, that is, US20100017145, US20110091924, US20080305962, US20100004871, US20100009401, US6300136 and US20040039553. The patents US20100017145, US20110091924, US20100009401 and US6300136 are about assessing cancer status by amino acid concentration detection. It could help assess the risk of developing tumors by analyzing the amino acid concentration contained in the blood. The other three patents US20080305962, US20100004871 and US20040039553 are on tumor-associated biomarkers. Also, one of the technology frontiers in development period was regarding diagnostic biomarkers. The seven patents with the highest degree centrality were all concerning tumor diagnosis in this period. Cancer is one of the causes of death around the world, which threatens human health and quality of life seriously. Early diagnosis and treatment could prolong survival time and improve quality of life [62].

The patent with the highest betweenness centrality and closeness centrality was the same one, that is, US4196265, concerning antibody culture. One of the technology frontiers in the development period was regarding to pathological diagnosis antibody. The field of pathological diagnosis could be divided into four sub-fields, namely histopathology, cytopathology, IHC pathology and molecular pathology. IHC pathology utilizes the principle of antigen-antibody specific binding to locate and qualitatively detect specific antigens or antibodies in tissues and cells, which has certain advantages over other techniques, and could form a good complementary relationship with related techniques. As the technology frontiers changed over time, the technology frontier of pathological diagnosis antibody was the continuation of the development of IHC [65]. The pathological diagnosis antibody played an important role in the evolution and development of technology, and also could result in more rapid diffusion of knowledge with other patents in its field.

#### 3.5.3 The high centrality patents in the rapid growth period (2016–2021)

In the rapid growth period (2016–2021), there was only one patent with the highest degree centrality, that is, US20170091937, which is about a computer-implemented method or DP enabled ML system, for predicting the risk of cancer recurrence in early stage of breast cancer patients, involving the technology frontier of “computational pathology” in this period. The patent with the highest betweenness centrality and closeness centrality was the same one, that is, US20180253591, which predicts cancer progression, by using a digitized pathology image of a region of tissue demonstrating cancerous pathology. It was related to the technology frontier of “evaluation and prediction models for pathological diagnosis” in this period.

The digitization of pathological slices has given rise to the possibility of building a large-scale DP library, the most famous of which is The Cancer Genome Atlas (TCGA), where researchers could freely obtain pathological images with labeled information and corresponding clinical, prognostic, and genomic information, which excites enthusiasm for the application of AI in DP and oncology research. ML, particularly DL, has enabled rapid advances in computational pathology [4], which could deliver computer models with image recognition that match or outperform human experts, and it is expected to improve the accuracy of histomorphological evaluation in pathological diagnosis. Systematic computational pathology research initiatives has been presented to accelerate the quantitative assessment of both morphological patterns and biomarker expression in histopathology [81].

## 4. Discussion

### 4.1 The evolution path of AI-assisted pathology

There were 1704 patents in the field of AI-assisted pathology, based on the data sources and search strategy. This field had received widespread attention, growing rapidly in recent years. Regression analysis showed that the patent applications would reach 511 in 2021 and 1040 in 2025, respectively. The field of AI-assisted pathology experienced three development periods, that is, the budding period (1992–2000), the development period (2001–2015), and the rapid growth period (2016–2021), with a different number of technology frontiers in each period. There were only 2 technology frontiers in the budding period, 5 ones in the development period, and 6 ones in the rapid development period. With the continuous development of science and technology, the number of technology frontiers had gradually increased. The development of AI had brought more possibilities to the field of pathology, the technology development in this field had spread to more directions, and the technology frontiers had become more and more extensive (Table 5). As was mentioned above, the patent with a higher degree centrality has greater influence and importance than others. In the budding period, there were 3 patents with the highest centrality, involving the technology frontier of IHC, mainly around the preparation and measurement methods of antibodies. In the early stage of technology development, a clear technology direction had not yet been formed. With the continuous technology development and the continuous expansion of application fields, the number of patents with high degree centrality would gradually increase, indicating that the technology was spreading to more fields. There were 7 patents with the highest centrality in the development period, mainly involving cancer evaluation and tumor biomarkers. During this period, a large number of technology development was focused on tumor diagnosis, and the field had been greatly developed. Cancer evaluation includes an evaluation method of cancer state and type, an evaluation device, etc. Tumor biomarkers are mainly based on biomarkers for diagnosis, treatment, and prediction. The research directions were broad, and the core technology in the field of AI-assisted pathology had not yet been formed. In the rapid development period, there was only one patent with the highest centrality, mainly involving computational pathology, which indicated that a relatively clear technology center had been formed in this field, and the direction of technology development was further focused. The most important advantage of the computational pathology is to reduce errors in diagnosis and classification. Computational pathology has the potential to transform the traditional core functions of pathology, not just growing sub-segments such as DP, molecular pathology, and pathology informatics, with the purpose of improving diagnostic accuracy, optimizing patient care, and reducing costs [82]. As the rapid technology advancement drives individualized precision medicine, computational pathology is a critical factor in achieving this goal [83]. Consequently, it could also be seen from the centrality that the core technology of AI-assisted pathology had also experienced a process from germination to divergence and then to concentration.

**Table 5:** The technology frontiers in each period.

Budding period 1990–2000	Development period 2001–2015	Rapid growth period 2016–2021
• Systems and methods for image data processing in computerized tomography (CT) • Immunohistochemistry (IHC)	**•** Spectral analysis methods of biomacromolecules **•** Pathological informationsystem **•** Diagnostic biomarkers **•** Molecular pathology diagnosis **•** Pathological diagnosis antibody	**•** Digital pathology (DP) **•** Deep Learning Algorithms—Convolutional Neural Networks (CNN) **•** Disease prediction models **•** Computational pathology **•** Pathological image analysis method **•** Intelligent pathological system

With the continuous improvement of various AI models and algorithms, the development of pathology presents more diverse, and the cross-integration of technology frontiers is becoming more and more significant. AI could solve the problems of data reproducibility and analysis complexity in pathology, through extracting information or features beyond human visual perception through algorithm training. The success of these AI-based approaches is inseparable from the support of algorithms, as well as the quantity and quality of data. As a branch of AI, ML could perform tasks without explicit programming instructions, discover hidden relationships between data, and perform data analysis. The wide application of ML methods in the field of pathology makes the technology direction appear diversified [2]. Commonly used ML methods have their own advantages and disadvantages. For example, logistic regression is easy to implement and interpret, with fast calculation speed and low memory usage. However, when the number of training samples is large, the performance of logistic regression is not good, and the phenomenon of under-fitting is prone to occur. Also, for nonlinear features, they are needed to be conversed. Compared with logistic regression, SVM could solve nonlinear problems, and it is a classifier that could be used directly without modification, getting a lower error rate. Yet, SVM is difficult to implement for large data training samples, and also difficult to solve the multi-classification problem. Compared to other algorithms, DT is easy to understand and explain, and rules could be easily extracted. It is able to produce feasible and effective results on large data sources in a relatively short period of time. While, it also has some disadvantages. Overfitting is prone to occur, and it is easy to ignore the correlation of attributes in the data set. RF is an ensemble algorithm composed of DT. Its advantages include fast training speed, easy parallel methods, simple implementation, etc., but it has been proved to be overfitting on some noisy classification or regression problem. NB is a simple yet powerful predictive modeling algorithm. It performs well on small-scale data, which could handle multiple classification tasks, and is suitable for incremental training. At the same, it also has shortcomings such as the need to calculate a priori probability, an error rate in classification decisions, being sensitive to the expression of input data. These traditional ML algorithms have been widely used in information classification (such as disease classification, cancer patient classification), image segmentation, disease prediction, etc., which could help enhance the reliability, performance, predictability, and accuracy of diagnostic systems for many diseases. Obviously, ML has certain limitations. In the future, it could be considered to develop new models based on various ML methods, in order to play to their respective strengths. For example, a study of University of Pennsylvania combined four ML algorithms, that is, RF, MLP, LightGBM, and XGBoost, to accurately predict the range of motion of lung tumors, and achieve precise and dynamic delineation of the target area [84]. The emergence of DL algorithms has led to better implementation of ML, and also the technology direction of pathology has been concentrated. The DL algorithms are constantly developing. There is no doubt that with the continuous improvement of algorithms, the pathology will present a new technology diversification trend.

IHC was an important technology frontier in the budding period (1992–2000), which is a commonly used technique and means in clinical pathological diagnosis. Since the 1970s, IHC has been used in pathological diagnosis, which has a huge impact on the diagnosis, classification and prognosis of tumors. Also, it expands people’s understanding of various diseases and tumor formation process, and improves the level of pathological diagnosis and research. Diagnostic biomarkers was one of the technology frontiers in the development period (2001–2015), which promotes the further development and progress of IHC. IHC is a routine pathological auxiliary diagnosis technology, which has been used to characterize biomarkers, such as programmed cell death-ligand 1 (PD-L1) [70]. PD-L1 is a biomarker of great interest, which is used to stratify cancer patients who may benefit from checkpoint inhibitor therapy. Currently, DL is used for IHC image analysis, including PD-L1, which greatly improves the accuracy. For example, DL can be used to identify and distinguish positive or negative tumor cells, as well as positive or negative inflammatory cells in PD-L1 imaging of lung cancer [85]. Hence, at the end of 2020, Peking University started a clinical trial (NCT04695015, Title: Research of pathological imaging diagnosis of ocular tumors based on new artificial intelligence algorithm), the purpose of which is to construct standardized digital ocular tumors with biomarkers pathology image database. The outcome measure is to compare the diagnostic accuracy of OPAL and IHC for melanoma and other tumors. AI has greatly promoted the development of tumor pathological diagnosis, which could visualize and quantify certain features in pathological sections and provide in-depth support for clinical decision-making.

Also, the application of AI in diagnostic biomarkers and molecular pathological diagnosis had further promoted tumor pathological diagnosis, both of which were the technology frontier in the development period (2001–2015). The patent analysis found that, the technology topics for biomarkers about AI-assisted pathology were mainly including tumor, DP, image analysis, pattern recognition, ML, etc. Obviously, there was cross-fusion between biomarkers and many other technology frontiers. Recently, patent applications for biomarkers have increased year by year. Tumor biomarker patents were the most, mainly for ovarian cancer (i.e., WO2011034596-A1), lung cancer (i.e., WO2015066564-A1), prostate cancer (i.e., US6025128-A) and others. Quantitative assessment of tumor biomarkers is a main content of precise pathological diagnosis. Pathologists have great advantages in qualitative interpretation of tumor biomarkers, but accurate quantification is subject to greater subjectivity, greater variability, and poor repeatability. It is more difficult for more complex quantitative markers such as multiple markers, while relying on AI-assisted computer image analysis has advantages. Pathological diagnosis is a necessary condition for tumor treatment decision-making. It could show the diverse forms of disease progression, and could reflect the individual status of patients, ability to receive treatment, response to treatment, and clinical outcomes. For example, The First Affiliated Hospital of Zhengzhou University and Sun Yat-sen University launched a clinical trial in 2017 (NCT04217044, Title: Histopathology images based prediction of molecular pathology in glioma using artificial intelligence), in order to construct and refine histopathology image based algorithms that are able to predict molecular pathology or subgroups of gliomas. AI is able to visualize and quantify certain features in pathological slides, providing deep support for clinical decision-making. There was a solution developed by Path XL Ltd and subsequently by Philips [86], which has been used for automated analysis and annotation of hematoxylin-eosin (H&E) tissue samples, to identify the boundary of the tumor and precisely measure tissue cellularity, as well as tumor cell content. It shows high levels of performance in lung cancer, and has been expanded to automatically identify tumor in colorectal, melanoma, breast, and prostate tissue section, providing an objective tissue quality evaluation for molecular pathology in solid tumors. A successful AI-based diagnosis assessment and prediction model for tumor pathology requires the cooperation of pathologists and oncologists. Pathologists provide knowledge bases for algorithm scientists involved in the design and development of AI algorithms, and guide developers to master specific attributes of pathological images, who train algorithms by annotation and segmentation of cells, tissue types, biological structures, or regions of interest. During the verification process, pathologists are also required to provide diagnostic references or standards, which are compared with ML algorithms, in order to promote the maturity of the algorithms. Oncologists provide research directions in application scenarios and perform clinical validation of algorithms. Eventually, pathological AI will be used to explore tumorigenesis and tumor evolution, and the research results will help pathologists and oncologists.

AI-assisted pathology is currently in a rapid development period, and DL has greatly facilitated the development of DP, computational pathology and other technology frontiers, all of which are concerning the study of algorithms. The continuous improvement and development of algorithms have the potential to not only improve the sensitivity and accuracy of the diagnosis, but also shorten the turnaround time. DL techniques are currently considered the state-of-the-art for image analysis. The average performance of DL models on classification tasks is better than traditional ML models. DL algorithms-CNN is one of the technology frontiers identified in our study, and there are many patent applications, having developed rapidly and attracted much attention in recent years. More than 40% of patents in the field of AI-assisted pathology are in regard to DL, the technology topics of which mainly involving CNN, network model, image processing, image segmentation, feature extraction, as well as training set, data set, etc. Among them, CNN-related patents are the most, accounting for about 1/3 of DL patents, which also confirms our research results that CNN was one of the technology frontiers of AI-assisted pathology and also a current research hotspot. The most commonly used DL model is the CNN, which has been applied to image analysis since the 1990s. As of July 20, 2022, a total of 109 clinical trials with respect to AI-assisted pathology had been retrieved in the ClinicalTrials.gov (https://clinicaltrials.gov/), including 13 ones regarding DL. There was one CNN-related clinical trial (NCT03822390, Title: Diagnostic performance of a convolutional neural network for diminutive colorectal polyp recognition), which was started by academic medical center of University of Amsterdam in October 2018. Computer aided diagnosis (CAD) based on CNN may facilitate endoscopists in diminutive polyp differentiation. The purpose of this clinical trial is to develop a CAD-CNN system, differentiating diminutive polyps during colonoscopy with high accuracy, and to compare the performance between this system and endoscopists, with the histopathology as the gold standard. CNN is a supervised learning algorithm, especially suitable for solving image classification problems. The emergence of large-scale labeled data such as ImageNet and the rapid improvement of GPU computing performance have led to a rapid explosion of research on CNN. The Google LLC has applied some patents pertaining to CNN (i.e. WO2021021329-A1, US2020285908-A1), which identify and analyze medical images to predict disease states or classify diseases by means of training DL model, such as CNN detection model. Moreover, Google LLC, with Shaare Zedek Medical Center, started a clinical trial (NCT04693078, Title: Detection of colonic polyps via a large scale artificial intelligence (AI) system) in May 2020, and the investigators proposed a new polyp detection system based on DL, which can alert the presence and location of polyps in real-time during a colonoscopy. Apparently, Google LLC has both patent applications and clinical trials in the field of AI-assisted pathology. It is expected that the related products will be launched in the future. AI has greatly promoted the development of pathology, especially DP, which has attracted widespread attention all over the world. In recent years, scientific research and technology development have been very active, not only with a large number of paper publications in DP, but also increasing patent applications significantly year by year. There were many high-quality reviews on AI in DP. One of them is a systematic review of the patents (Title: Current trend of artificial intelligence patents in digital pathology: a systematic evaluation of the patent landscape) we mentioned earlier, published in May, 2022, which concludes that the field of AI in DP grows rapidly according to the increasing patent numbers, and foresees a continuous growth in the number of patent applications internationally [20]. Furthermore, Nature published an opinion article (Title: Artificial intelligence in digital pathology—new tools for diagnosis and precision oncology) in 2019 [8], with 334 citations, which critically evaluates various AI-based computational approaches for DP, discusses some of the challenges pertaining the use of AI, and presents potential future opportunities for precision oncology. There is also a review, with 20 citations, published in 2021 in regard to DP and AI in translational medicine and clinical practice [70], which discusses the opportunities and limitations in AI-based methods, how considering the advances of DP and AI in translational medicine, and the challenges being confronted with if utilizing in clinical practice. From the published scientific papers, DP was a very concerned research hotspot. The patent applications of DP were also very active in last few years, nearly 1 in 10 patent applications in the field of AI-assisted pathology were concerning DP, and the technology topics were made of WSI, digital image, image processing, DL and so on. It could also be seen from the distribution of technology topics that the technology cross-integration has become very common. DP is not only fruitful in scientific research and technology development, but also has products on the market. From our patent analysis, it was found that Leica Biosystems laid out more patents in DP. Besides, it has also three categories of DP-related products, which are slide scanners & solutions (Aperio GT 450, Aperio CS2, Aperio VERSA, Aperio LV1), management & integration software (Aperio eSlide Manager, Aperio ImageScope) and image analysis (algorithm menu including IHC, Immunofluorescence, ISH & FISH). Leica Biosystems has mature products of hardware, software and algorithms of DP. There is no doubt that DP is a very concerned technology frontier for AI-assisted pathology. With the continuous development and progress of AI, it could be predicted that there will be more patents to be deployed around hardware, software and algorithms of DP, and product upgrades will continue. Computational pathology has recently shown great promise, the most important advantage of which is to reduce errors in diagnosis and classification. It could offer a better-integrated solution to WSI, multi-omics data, and clinical informatics. Professor Thomas Fuchs, known as the “Father of Computational Pathology”, the first one giving definition of computational pathology, who discussed the development prospects of computational pathology in histopathology, clinical applications and decision support in the paper “Computational pathology: challenges and promises for tissue analysis”, published on the journal of “Computerized Medical Imaging and Graphics” in 2011 [87]. In recent years, his team has also begun to explore the application of DL in the field of computational pathology. Many scientific papers have been published in regard to computational pathology, most of which are on oncology. Cancer analysis is one of the very important application scenarios of computational pathology. The continuous improvement and development of algorithms promote the progress of computational pathology, which makes the classification and analysis of cancer images more accurate, such as breast cancer [88], gastric cancer [89], and colorectal cancer [90]. There are also many highly cited reviews. One of reviews was published in 2021 (Title: Artificial intelligence and computational pathology) [83], with 58 citations, which discusses the statistical methods, clinical applications, potential obstacles, and future directions of computational pathology. In recent years, patent applications for computational pathology have also increased year by year, and the technology topics are mainly around DL, image segmentation, model optimization, etc. The patent assignee with the most patent applications in computational pathology was PAIGE AI Inc. (i.e., WO2021183955-A1, US2021233251-A1, US2021209753-A1), the patents of which are mainly about processing images by ML models. PAIGE AI Inc. was founded by Thomas Fuchs, and his team is comprised of pathologists, AI researchers, and medical experts, with the purpose of seeking to transform the way of cancer diagnosis and guide treatment through technologies such as AI and computational pathology. One of its products is the PAIGE Guidance Engine, which is capable of performing tasks such as rapid diagnostic stratification, tumor detection, segmentation, treatment response and survival prediction. In 2019, the DP solution PAIGE.AI was granted breakthrough device designation by the FDA [8], and it was the first time awarded to an AI-based cancer diagnostic research and development company. The growing medical big data, including genomics, proteomics, informatics, and WSI, will be integrated together to bring a rapid development of AI-assisted computational pathology.

From the perspective of the technology frontier distribution in the field of AI-assisted pathology in recent years, apart from algorithm improvement, precise diagnosis will be one of the future research hotspots due to the development of DP and computational pathology. The accuracy and repeatability of diagnosis are continuously improved by improving algorithms, and intelligent diagnostic systems are developed by optimizing models, in order to realize the precise diagnosis. In addition, data standardization is also an important direction for future development. The core elements of the rapid development of AI are big data, algorithms and supercomputing. AI requires practice based on large amounts of data, so data quality directly determines the quality of learning outcomes. At present, there are some problems such as lack of uniform standards, uneven quality and hospital protection of medical data in various regions. Hence, data standardization is a huge challenge for future development.

### 4.2 Limitations

There were several limitations in our research, for example, only patent data were used. The patent analysis was based on all patent applications, no authorized patents had been isolated to do further research, and also the issue of patent maintenance had not been considered. Due to many patents lack of value any more, patent assignees would give up continuing to pay for maintenance. This was also an issue we needed to consider. Therefore, when analyzing the state of technology development, there may be certain deviations by just analyzing patent applications, which could not fully reflect the actual situations. Besides, the technology development is not only reflected by patents, but also in many applied researches, which should also be included in the technology research. Hence, our future technology research plans to analyze data from multiple sources, scientific papers involving applied research should be the subject of technology research along with patents, which would be more scientific, objective and reasonable.

Citation analysis is an important analytical method in bibliometrics, scientometrics, informetrics, and patentometrics. Patent citation analysis is one of the main means of patent analysis, which could be used to carry out technology assessment, identify key technologies, etc. Moreover, patent citations could reflect knowledge flow and technology transfer of a certain field. There was another limitation in our research, which was not taking into account the issue of citation motivation. There are two sources of patent citations, one from the inventors, and the other from the examiners, which are added during the examination process. Some studies had pointed out that the inventors would instinctively avoid the same or similar prior art, and the degree of correlation between patent documents cited by inventors and patent applications is much lower than that of citations added by examiners. Therefore, in order to conduct more scientific and objective research based on patent citations, it is necessary to classify and analyze the citations at first, and then chose appropriate patent citations scientifically and prudently for subsequent research.

## 5. Conclusions

This study used patent analysis to explore the research activities and reveal the current characteristics in the field of AI-assisted pathology. This field has received widespread attention, growing rapidly in recent years. The field of AI-assisted pathology experienced three development periods, they were the budding period (1992–2000), the development period (2001–2015), and the rapid growth period (2016–2021), with a different number of technology frontiers in each period. AI broadens the ideas for accurate pathological diagnosis, and breaks the bottleneck of technology and pathologist ability. Our research found that the current technology frontiers focused on DL algorithms, DP, computational pathology, with active paper publications and patent applications, as well as related products, most of which were the studies on oncology. With the continuous development of new algorithms, and the discovery of new biomarkers, DP and computational pathology have achieved remarkable results in the field of oncology. The research hotspots are focusing on image processing and algorithm improvement, which could achieve more accurate image segmentation and classification, and enhance the accuracy and efficiency of diagnosis. In recent years, with the rapid development of DL, computational pathology resulting from the collaborative application of DP and AI, and the more extensive intelligent pathology, which would definitely bring a better prospect of precise pathological diagnosis.

There were several limitations in our research. Firstly, only patent data were used for technology analysis. Patents cannot fully reflect the technology development, and the scientific papers related to applied research should also be included. Integrating data from multiple sources will make the research more comprehensive and scientific. Secondly, we adopted patent citation analysis, without taking into account the issue of citation motivation. There are two sources of patent citations, one from the inventors and the other from the examiners, with different citation motivations. In order to make more scientific and rational research, it is necessary to classify the citations, and chose appropriate patent citations for analysis.

AI-assisted pathology is developing rapidly, which has the potential to transform pathology through the application of accelerated workflows, and to improve diagnosis and patient clinical outcomes. Continued research is required in the future to develop new AI algorithms for medical applications and to improve existing ones. There will be more DP workflows to come. Based on many existing research results of precise pathological diagnosis, AI could match the performance of pathologists better, even exceed the existing capabilities and knowledge of pathologists in more possible areas. The close integration and complementary advantages of pathologists and AI will bring precise pathological diagnosis to a new level. The results of this study presented an overview of the characteristics of research status and trends of AI-assisted pathology, which could help readers broaden innovative ideas and discover new technology opportunities, and also served as important indicators for government policymaking.

## Supporting information

S1 File The patent information of each technology frontier. (XLSX)

S2 File The related clinical trials of some technology frontiers. (XLSX)

## Abbreviation

AI: artificial intelligence
ML: machine learning
NLP: natural language processing
DT: decision tree
NB: naive bayes
RF: random forest
SVM: support vector machine
MLP: multi-layer perceptron
DL: deep learning
GAN: generative adversarial network
RNN: recurrent neural networks
R-NN: recursive neural network
CNN: convolutional neural network
WSI: whole slide imaging
HMM: hidden markov models
MS: mass spectrometry
DP: digital pathology
DII: Derwent Innovation Index
DWPI: Derwent World Patent Index
DPCI: Derwent Patents Citation Index
DDA: Derwent Data Analyzer
SNA: social network analysis
IHC: immunohistochemistry
CT: computerized tomography
AD: Alzheimer’s disease
